# Parental Nonadherence to Health Policy Recommendations for Prevention of COVID-19 Transmission Among Children

**DOI:** 10.1001/jamanetworkopen.2023.1587

**Published:** 2023-03-06

**Authors:** Andrea Gurmankin Levy, Alistair Thorpe, Laura D. Scherer, Aaron M. Scherer, Jorie M. Butler, Holly Shoemaker, Angela Fagerlin

**Affiliations:** 1 Department of Social Science, Middlesex Community College, Middletown, Connecticut; 2 Department of Population Health Sciences, University of Utah Spencer Fox Eccles School of Medicine, Salt Lake City; 3 currently affiliated with Department of Applied Health Research, University College London, London, United Kingdom; 4 Division of Cardiology, University of Colorado School of Medicine, Aurora; 5 Veterans Affairs (VA) Denver Center for Innovation, Denver, Colorado; 6 Department of Internal Medicine, University of Iowa School of Medicine, Iowa City; 7 Division of Geriatrics, Departments of Biomedical Informatics and Internal Medicine, University of Utah Spencer Fox Eccles School of Medicine, Salt Lake City; 8 Salt Lake City VA Informatics Decision-Enhancement and Analytic Sciences (IDEAS) Center for Innovation, Salt Lake City, Utah; 9 Geriatrics Research, Education, and Clinical Center, VA Salt Lake City Health Care System, Salt Lake City, Utah

## Abstract

This survey study assesses whether parents had ever engaged in specific misrepresentation and nonadherence behaviors regarding public health measures for preventing COVID-19 transmission among children.

## Introduction

People are not always honest about their medical information^1^ or adherent to medical recommendations,^2^ including the public health measures (PHMs) against COVID-19 (eg, not reporting symptoms, breaking quarantine).^3^ During the COVID-19 pandemic, parents experienced greater increases in stress compared with nonparents due to additional child-related PHMs (eg, school closings, quarantine rules for children).^4^ We examined the prevalence of misrepresentations of and nonadherence to COVID-19–related PHMs by parents regarding their children (eg, breaking quarantine rules by sending their child to school so that the parent can work), their reasons, and associations of individual characteristics with these behaviors.

## Methods

This survey study recruited a national, nonprobability sample of US adults through Qualtrics for an online survey about COVID-19 experiences (participation, 1811 of 2260 [80.1%]) from December 8 to 23, 2021. The survey asked whether parents had ever engaged in 7 types of misrepresentation and nonadherence behaviors regarding COVID-19 PHMs for their children (Table 1) and reasons for these behaviors (Table 2). Additional methodological information is published elsewhere.^3^ The University of Utah Institutional Review Board deemed the study exempt and granted a waiver of informed consent owing to no risk or minimal risk to participants. The study followed the AAPOR reporting guideline.

**Table 1.** Frequency With Which Parents Engaged in Misrepresentation of and Nonadherence to COVID-19 Recommendations

Response	No./total No. (%) [95% CI]
Misrepresentation	
Did not mention that they thought or knew their child had COVID-19 to someone their child was with or about to be with in person^a^	63/263 (24.0) [19.2-29.5]
Said that their child was older than they actually were so the child could get vaccinated	56/578 (9.7) [7.5-12.4]
Said that their child was vaccinated for COVID-19 when they were not^b^	47/464 (10.1) [7.7-13.2]
Said that their child was not vaccinated when they were^c^	23/189 (12.2) [8.2-17.6]
Said that their child did not have to quarantine even though they were supposed to^d^	52/318 (16.4) [12.7- 20.8]
Nonadherence	
Avoided getting child tested when they thought their child might have COVID-19^e^	44/227 (19.4) [14.8- 25.0]
Allowed their child to break quarantine rules^d^	67/318 (21.1) [16.9-25.9]

^a^ Among those who thought or knew their child had COVID-19.

^b^ Among those whose child was not vaccinated.

^c^ Among those whose child was vaccinated.

^d^ Among those whose child was told to quarantine.

^e^ Among those who thought their child might have COVID-19.

**Table 2.** Reasons Participants Indicated for Misrepresentation of and Nonadherence to COVID-19 Recommendations

Reason	No. (%) of parents						
	Exposure	Quarantine		Vaccination			Testing
	Did not mention that they thought or knew child had COVID-19 to someone child was with or about to be with in person (n = 63)	Said did not have to quarantine even though were supposed to (n = 52)	Broke quarantine rules (n = 67)	Said child was older than actually was so could get COVID-19 vaccine (n = 56)	Said vaccinated when not vaccinated (n = 47)	Said not vaccinated when vaccinated (n = 23)	Avoided getting tested when thought might have COVID-19 (n = 44)
I wanted to exercise my freedom to do what I want with my child	33 (52.4)	19 (36.5)	32 (47.8)	37 (66.1)	31 (66.0)	NA	22 (50.0)
My child did not feel very sick	30 (47.6)	20 (38.5)	28 (41.8)	NA	NA	NA	15 (34.1)
I wanted my child’s life to feel “normal” (ie, how they felt before the COVID-19 pandemic began)	29 (46.0)	22 (42.3)	30 (44.8)	NA	28 (59.6)	NA	NA
I did not want to miss an event or other fun activity to stay home	28 (44.4)	18 (34.6)	21 (31.3)	NA	NA	NA	NA
I could not miss important nonwork responsibilities to stay home (eg, get groceries, care for loved ones)	27 (42.9)	21 (40.4)	29 (43.3)	NA	NA	NA	NA
I did not think my child really had COVID-19	27 (42.9)	22 (42.3)	28 (41.8)	NA	NA	NA	14 (31.8)
I did not want my child to miss school to stay home	27 (42.9)	19 (36.5)	20 (29.9)	NA	NA	NA	NA
I was following guidance from a public figure that I trust (eg, politicians, scientists, people on the news, celebrities)	25 (39.7)	20 (38.5)	19 (28.4)	NA	24 (51.1)	15 (65.2)	16 (36.4)
I did not want someone to judge or think badly of me or my child	25 (39.7)	20 (38.5)	23 (34.3)	NA	21 (44.7)	13 (56.5)	13 (29.5)
I did not want my child to miss an event or fun activity to stay home	25 (39.7)	21 (40.4)	22 (32.8)	NA	NA	NA	NA
I did not want my child to miss important activities to stay home (eg, music, sports, clubs)	24 (38.1)	24 (46.2)	26 (38.8)	NA	NA	NA	NA
I was confused about the rules for quarantine	23 (36.5)	13 (25.0)	16 (23.9)	NA	NA	NA	NA
My child was bored or lonely	22 (34.9)	16 (30.8)	24 (35.8)	NA	NA	NA	NA
I could not miss work to stay home	22 (34.9)	17 (32.7)	22 (32.8)	NA	NA	NA	NA
I did not want them to be angry at me or my child for exposing them	22 (34.9)	25 (48.1)	NA	NA	NA	NA	NA
I did not want certain people to know	21 (33.3)	18 (34.6)	16 (23.9)	NA	22 (46.8)	8 (34.8)	14 (31.8)
It is no one else’s business	20 (31.7)	16 (30.8)	30 (44.8)	NA	19 (40.4)	10 (43.5)	18 (40.9)
I did not think it mattered	19 (30.2)	20 (38.5)	22 (32.8)	NA	18 (38.3)	9 (39.1)	14 (31.8)
I did not think COVID-19 was a big deal	18 (28.6)	20 (38.5)	14 (20.9)	NA	15 (31.9	NA	12 (27.3)
I did not think COVID-19 was real	16 (25.4)	16 (30.8)	18 (26.9)	NA	16 (34.0)	NA	9 (20.5)
I wanted my child vaccinated to lower their risk of COVID-19 in time for school or camp	NA	NA	NA	36 (64.3)	NA	NA	NA
I wanted my child vaccinated to lower their risk of COVID-19 in time for a trip or a visit with family or friends	NA	NA	NA	32 (57.1)	NA	NA	NA
I wanted my child vaccinated to lower their risk of COVID-19 in general	NA	NA	NA	30 (53.6)	NA	NA	NA
I wanted my child vaccinated to lower their risk of COVID-19 in time for an event or activity that they were participating in	NA	NA	NA	28 (50.0)	NA	NA	NA
I wanted my child to be able to do something where being vaccinated was required (eg, a special event, get together with friends or family who were vaccinated, etc)	NA	NA	NA	NA	28 (59.6)	NA	NA
I did not want to have to deal with the consequences of a test showing that my child had COVID-19 (eg, my family would have to quarantine, I would have to miss work, my child would miss school, etc)	NA	NA	NA	NA	NA	NA	16 (36.4)
My child and/or I were worried it would hurt or be uncomfortable to get tested	NA	NA	NA	NA	NA	NA	24 (54.5)
I wanted to keep COVID-19 rates low in my area so public health measures were not put in place (eg, closing schools, mask mandates)	NA	NA	NA	NA	NA	NA	15 (34.1)
I did not want the government to have my child’s personal information	NA	NA	NA	NA	NA	NA	14 (31.8)
I thought I could not afford the cost of getting tested	NA	NA	NA	NA	NA	NA	16 (36.4)
I did not have time to get my child tested	NA	NA	NA	NA	NA	NA	14 (31.8)
I did not know how or where to get my child tested	NA	NA	NA	NA	NA	NA	11 (25.0)

Abbreviation: NA, not applicable.

The final sample consisted of 1733 US adults. The analyses included the 580 parent participants (33.5%) who had children younger than 18 years living with them during the pandemic. Race and ethnicity data were collected because COVID-19 and public health measures disproportionately impacted individuals from underserved populations. Descriptive statistics examined the prevalence of and reasons for misrepresentation and nonadherence, and multiple logistic regression was used to explore potential associated characteristics. Significance was set at 2-sided α = .05 with *P* values adjusted using Holm-Bonferroni correction. Analyses were performed using R Studio, version 1.4.1106 (R Program for Statistical Computing).

## Results

Among the 580 participants, the mean (SD) age was 35.9 (8.8) years; 403 (70.2%) identified as women compared with 171 (29.5%) men and 6 (1.0%) other or missing. In terms of race and ethnicity, 80 participants (13.8%) were Hispanic, 5 (0.9%) non-Hispanic American Indian or Alaskan Native, 14 (2.4%) non-Hispanic Asian, 86 (14.8%) non-Hispanic Black, 389 (67.1%) non-Hispanic White, 5 (0.9%) more than 1 race, and 1 (0.2%) White with no ethnicity specified.

One hundred fifty participants (25.9%) reported misrepresentation and/or nonadherence in at least 1 of 7 behaviors; the most common behaviors were not telling someone who was with their child that they thought or knew their child had COVID-19 (63 of 263 [24.0%]) and allowing their child to break quarantine rules (67 of 318 [21.1%]) (Table 1). The most common reason was wanting to exercise personal freedom as a parent. Additional reasons included wanting their child’s life to feel normal and not being able to miss work or other responsibilities to stay home (Table 2). In an exploratory multiple logistic regression, no characteristics (eg, education, religiosity) were associated with misrepresentation or nonadherence.

## Discussion

In this survey study of US parents, one-quarter engaged in misrepresentation or nonadherence regarding PHMs for their children. The most common reason was to preserve parental autonomy. Additional reasons included wanting to resume a normal life for their child and the inability to miss work or other responsibilities, among other reasons.

These results suggest that some PHMs implemented to limit the spread of COVID-19 may have been compromised due to misrepresentation and nonadherence by parents on behalf of their children, contributing to COVID-19–related morbidity and mortality. In addition, some children appear to have received a vaccine that was not fully tested and approved in their age group.

Study limitations include the nonprobability sample, the exploratory nature of the regression model, and the possibility that social desirability motivated underreporting, potentially resulting in an underestimate of the prevalence of misrepresentation and nonadherence. Nevertheless, our findings suggest a serious public health challenge in the immediate context of the COVID-19 pandemic, including future waves affecting weary parents, as well as future infectious disease outbreaks. Further work is needed to identify groups at highest risk of misrepresentation and nonadherence, to address parents’ concerns that were identified as reasons for these behaviors (eg, desire for autonomy), and to implement better support mechanisms for parents (eg, paid sick leave for family illness) during such crises so that misrepresentation and nonadherence feel less necessary.

## Notes

Supplement.
Data Sharing Statement
