# Supplementary material for: Parental Nonadherence to Health Policy Recommendations for Prevention of COVID-19 Transmission Among Children
Source: JAMA Netw Open. 2023 Mar 6;6(3):e231587. doi: 10.1001/jamanetworkopen.2023.1587 (PMC9989896; doi:10.1001/jamanetworkopen.2023.1587)
Supplement: Supplement. — Data Sharing Statement [file jamanetwopen-e231587-s001.pdf]

Data Sharing Statement

Levy. Parental Nonadherence to Health Policy Recommendations for Prevention of COVID-19 Transmission Among Children. *JAMA Netw Open*. Published March 06, 2023.
doi:10.1001/jamanetworkopen.2023.1587

Data
Data available: Yes
Data types: Deidentified participant data, Data dictionary
How to access data: andylevy4@gmail.com
When available: beginning date: 06-01-2023

Supporting Documents
Document types: None

Additional Information
Who can access the data: Anyone requesting the data.
Types of analyses: Any purpose.
Mechanisms of data availability: Without investigator support.
